# Endoscopic Nasobiliary Drainage in the Management of Acute Cholangitis: An Experience in 143 Patients

**DOI:** 10.1155/DTE.3.161

**Published:** 1997

**Authors:** M. K. Goenka, D. K. Bhasin, R. Kochhar, B. Nagi, U. Rungta, K. Das, K. Singh

**Affiliations:** 1 Sections of Radiology and Clinical Gastroenterology Department of Gastroenterology Postgraduate Institute of Medical Education and Research Chandigar 160012 India; 2 Eko Endoscopy Centre 54 JLN Road Calcutta 700 071 India

## Abstract

Acute cholangitis is associated with a high mortality and morbidity and often requires drainage of the obstructed biliary system. The purpose of this study was to evaluate the usefulness and safety of endoscopic nasobiliary drainage in the treatment and prevention of acute cholangitis due to diverse etiology. During a 32-month period, 143 patients (67 males, 76 females) with age range of 15 to 84 years underwent urgent fluoroscopy guided endoscopic nasobiliary drainage using a 7 Fr catheter either to treat acute cholangitis not responding to antibiotics (group A, n = 116) or to prevent its development following endoscopic retrograde cholangiography performed in an obstructed biliary system (group B, n = 27). Underlying etiology included bile duct stones (92), malignant biliary obstruction (34), choledochal cyst (4), chronic pancreatitis (4), ruptured hydatid cyst (3), portal hypertensive cholangiopathy (3) and liver abscess (3). Endoscopic nasobiliary drainage was performed successfully in 129 patients (90.2%). Cholangitis improved within 1 to 3 days (in group A) or did not develop (in Group B) in 125 patients (96.7%) with successful endoscopic nasobiliary drainage. Two patients however required additional drainage by percutaneous transhepatic route, while two died inspite of effective endoscopic drainage. Of the 14 patients (9.8%) with failed endoscopic drainage, 9 were managed by surgical decompression or percutaneous transhepatic drainage, 3 died of septicemia. Endoscopic nasobiliary drainage is a safe and effective method to treat patients with acute cholangitis as well as to prevent its development following cholangiography performed in an obstructed biliary system.


*Diagnostic and Therapeutic Endoscopy*, 1997, Vol. 3, pp. 161–170
Reprints available directly from the publisher
Photocopying permitted by license only

© 1997 OPA (Overseas Publishers Association)
Amsterdam B.V. Published in The Netherlands
by Harwood Academic Publishers
Printed in Singapore

### Endoscopic Nasobiliary Drainage in the Management of Acute Cholangitis: An Experience in 143 Patients

M.K. GOENKA,*,† D.K. BHASIN, R. KOCHHAR, B. NAGI,
U. RUNGTA, K. DAS and K. SINGH

*Sections of Radiology and Clinical Gastroenterology, Department of Gastroenterology, Postgraduate Institute of Medical Education and Research, Chandigar 160012, India*

*(Received 8 May 1996; Revised 20 June 1996; In final form 8 July 1996)*

**Acute cholangitis is associated with a high mortality and morbidity and often requires drainage of the obstructed biliary system. The purpose of this study was to evaluate the usefulness and safety of endoscopic nasobiliary drainage in the treatment and prevention of acute cholangitis due to diverse etiology. During a 32-month period, 143 patients (67 males, 76 females) with age range of 15 to 84 years underwent urgent fluoroscopy guided endoscopic nasobiliary drainage using a 7 Fr catheter either to treat acute cholangitis not responding to antibiotics (group A, n = 116) or to prevent its development following endoscopic retrograde cholangiography performed in an obstructed biliary system (group B, n = 27). Underlying etiology included bile duct stones (92), malignant biliary obstruction (34), choledochal cyst (4), chronic pancreatitis (4), ruptured hydatid cyst (3), portal hypertensive cholangiopathy (3) and liver abscess (3). Endoscopic nasobiliary drainage was performed successfully in 129 patients (90.2%). Cholangitis improved within 1 to 3 days (in group A) or did not develop (in Group B) in 125 patients (96.7%) with successful endoscopic nasobiliary drainage. Two patients however required additional drainage by percutaneous transhepatic route, while two died inspite of effective endoscopic drainage. Of the 14 patients (9.8%) with failed endoscopic drainage, 9 were managed by surgical decompression or percutaneous transhepatic drainage, 3 died of septicemia. Endoscopic nasobiliary drainage is a safe and effective method to treat patients with acute cholangitis as well as to prevent its development following cholangiography performed in an obstructed biliary system.**

*Keywords:* Bile ducts stenosis, bile ducts prosthesis, cholangitis, endoscopic sphincterotomy

*Corresponding Author: Tel: 0091-33-3341193 Fax: 0091-33-2428098.
†Present address: Eko Endoscopy Centre, 54 JLN Road, Calcutta 700 071, India.

### INTRODUCTION

Acute cholangitis often complicates obstructed biliary system due to choledocholithiasis, biliary stricture and less commonly due to malignancy of pancreaticobiliary region. Risk of acute cholangitis is increased if radiographic contrast material is injected into an obstructed biliary system[1]. While all such patients require antibiotic treatment, a significant proportion do need drainage of the obstructed system[1]. The drainage was conventionally done by surgery[2–4], but over the last decade, radiological[5–7] and endoscopic techniques[1,8–14] for drainage have been introduced. Endoscopic drainage can be achieved by endoscopic sphincterotomy, biliary stenting or nasobiliary catheter drainage[1,8–14]. While a number of studies have shown endotherapy of cholangitis to be a safe and useful technique, these are mostly based on a limited number of patients[8,10–13], and most have included only patients with choledocholithiasis[9–14]. We report our experience of 143 patients treated by endoscopic nasobiliary drainage to treat acute cholangitis or to prevent its development in patients with obstructed biliary system due to a diverse underlying etiology.

### MATERIAL AND METHODS

Between May, 1993 and December, 1995, 143 patients underwent endoscopic nasobiliary drainage (ENBD) either to treat acute cholangitis (Group A, therapeutic group, n = 116) or to prevent the risk of cholangitis following endoscopic retrograde cholangiopancreatography (ERCP) performed in an obstructed biliary system with failure to relieve the obstruction endoscopically (Group B, prophylactic group, n = 27).

All patients underwent clinical evaluation, laboratory investigations including liver function tests, renal parameters and serum electrolytes. An abdominal ultrasonogram was also performed in all patients. Initial treatment of patients with features of cholangitis (Group A) included intravenous antibiotic (ciprofloxacin or piperacillin), parenteral fluid and injections of vitamin K, while those without cholangitis (Group B) were started on oral ciprofloxacin 12–24 hours prior to ERCP. Four patients in Group A having hyperkalemia due to renal failure secondary to cholangitis and septicemia received dialysis prior to ERCP. ERCP was performed in Group A patients only after a sub-optimum response to medical therapy instituted for a period of 1 to 3 days.

ERCP was performed under fluoroscopic guidance using a side-viewing duodenoscope (JF-1T or JF-IT20, Olympus or FD34X, Ashai Optical) after intravenous diazepam (5–10 mg) and/or hyoscine N-butyl bromide (20–60 mg). A small amount of 60% meglumine iothalamate was used for diagnostic ERCP. Endoscopic sphincterotomy was performed in only 11 patients as a pre-requisite for attempted stone extraction. A 0.035 inch guide-wire (Zebra, Microvasive, Boston Scientific, USA) was passed through the ERCP cannula and after positioning its tip proximal to the site of obstruction, the cannula was withdrawn. In patients with tight biliary stricture, a 6 Fr biliary dilator (Wilson Cook, Winston-Salem) was passed over the guide wire. Nasobiliary catheter (7 Fr, pig-tail) was threaded over the guide wire, passed through the biopsy channel of the endoscope and its tip positioned proximal to the site of obstruction or into the abscess cavity (in patients with liver abscess, hydatid cyst and choledochal cyst). Guide wire was then withdrawn followed by the endoscope. After ensuring a free flow of bile from the external end of ENBD catheter, the catheter was rerouted through the nose by a rail-road technique using a nasogastric tube. A cholangiogram was repeated through the ENBD catheter using 60% meglumine iothalamate to adequately visualise the biliary system. ENBD catheter, while in position, was irrigated once daily using 20 ml–60 ml of sterile normal saline.

Response to endoscopic drainage was monitored in terms of clinical improvement as well as changes in liver function tests. Patients were offered definitive therapy for the underlying etiology, once the cholangitis settled and patient stabilised.

TABLE I Etiology in patients undergoing endoscopic nasobiliary drainage

| Etiology | Group A (Therapeutic) n = 116 | Group (Prophylactic) n = 27 | Total n = 143 |
|---|---|---|---|
| **Benign (n=109)** | | | |
| Bile duct stones | 83 | 9*** | 92 |
| Choledochal cyst | 4 | – | 4 |
| Chronic pancreatitis with biliary stricture | 3 | 1 | 4 |
| Hydatid cyst with biliary rupture | 3 | – | 3 |
| Portal cholangiopathy | 3 | – | 3 |
| Cholangitic abscess | 3 | – | 3 |
| **Malignant (n = 34)** | | | |
| Ampullary carcinoma | 7 | 4 | 11 |
| Malignant biliary structure* | 8** | 10 | 18 |
| Pancreatic head cancer | 2 | 3 | 5 |

*Includes gall bladder cancer with bile duct infiltration as well as cholangiocarcinoma
**Two of these patients ahd cholangitis following occlusion of biliary stent
***Failed stone extraction because of large stones (4), associated stricture (2), abnormal coagulogram (1)

### RESULTS

There were 67 men and 76 women with an age range of 15 to 84 years. Table I gives the underlying etiology of cholangitis or biliary obstruction needing ENBD. Among patients with cholangitis, common bile duct stones constituted the major proportion (n = 83, 71.6%) (Fig. 1), with 22 among them having ductal stones following cholecystectomy. Of the 9 patients with stone disease in prophylactic group, stone extraction was not possible because of large size of the stone in 4 patients, associated biliary stricture in 2, abnormal coagulogram in 1 and a technical failure in 2 patients. A total of 34 patients (17 each in group A and group B) had malignant etiology of bile duct obstruction (Fig. 2 and 3), two of them developed cholangitis following occlusion of plastic biliary stent placed earlier for inoperable gall bladder carcinoma infiltrating the bile duct. Four patients with cholangitis had choledochal cyst (Fig. 4), while 3 patients had hydatid cyst of liver with biliary communition with major bile duct shown at ERCP (Fig. 5). Nasobiliary catheters were placed with tip in the choledochal cyst and hydatid cyst respectively (Fig. 4 and 5). Three patients with portal hypertension due to extrahepatic portal venous obstruction presented with jaundice and cholangitis, ERCP showed biliary tract abnormalities including stricture (n = 2) (Fig. 6) or multiple filling defects due to intraductal varices (n = 1). These were categorised as portal hypertensive cholangiopathy and cholangitis treated by ENBD (Fig. 6).

Among Group A, history of jaundice was present in 108 (93.1%) patients, abdominal pain in 100 (86.2%) and fever in 103 (88.8%) while in Group B, 22 (81.5%) patients had jaundice and 19 (70.4%) had abdominal pain. Serum bilirubin was found to be raised in 114 patients (79.7%), range of serum bilirubin varying between 0.5 to 46 mg/dL with a mean (±SEM) of 11.2 (±1.2) mg/dL. Similarly serum alkaline phosphatase was abnormally high in 118 (82.5%) patients; the range among the total patient population being 4.1 to 104 King Armstrong Units. Abdominal ultrasonogram demonstrated dilatation of intrahepatic biliary radicles and/or common bile duct in 114 patients (79.7%), while bile duct stones could be visualised at ultrasonogram in 60 (65.2%) out of 92 patients

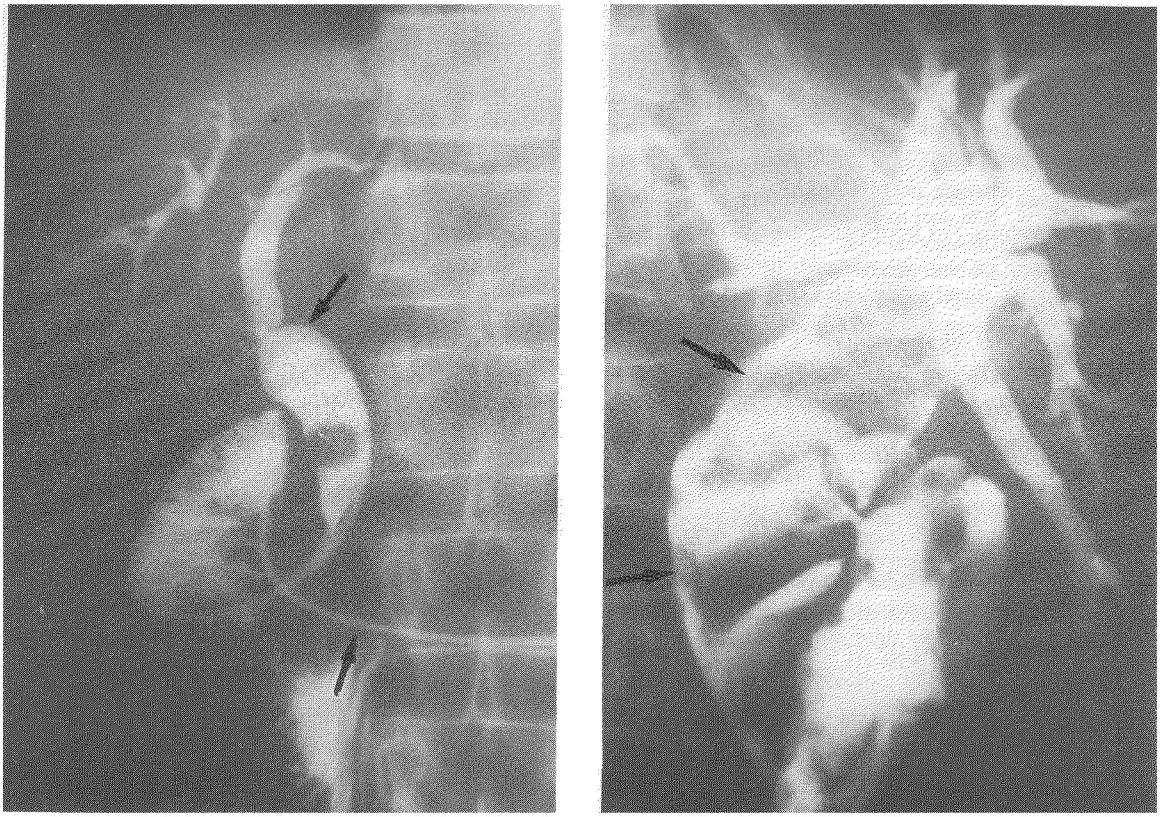

FIGURE 1 Two patients with common bile duct stone and cholangitis treated by endoscopic nasobiliary drainage (arrows) (a) Solitary stone, treated subsequently by endoscopic stone extraction (b) Multiple large stones, required surgery for stone removal after control of cholangitis.

diagnosed to have stone at ERCP. Twelve patients, all in group A had abscesses visualised at ultrasonogram, while 15 out of 34 patients with malignant biliary stricture had tumor shown at ultrasonogram.

Figure 7 shows the outcome of ENBD placement. Nasobiliary catheter could be positioned successfully in 129 patients (90.2%). Failure in 14 patients (malignant obstruction: 9, chronic pancreatitis: 1, stone disease: 4) was due to failure to cannulate the bile duct in 3 patients and failure to negotiate guide wire across the obstruction in 11 patients due to tight stricture (n = 8), multiple stones (n = 2) or a large stone (n = 1). Of the 8 patients with tight biliary stricture and failure to negotiate the guide wire, one had benign stricture due to chronic pancreatitis, two had ampullary carcinoma and five had high bile duct malignant stricture either due to infiltration from carcinoma of gall bladder or due to cholangiocarcinoma. While 5 of these patients with failed ENBD underwent percutaneous transhepatic biliary drainage, 4 underwent biliary tract surgery. Among the failed ENBD group, 3 patients died due to uncontrolled cholangitis and septicemia, 1 of these inspite of percutaneous drainage.

Of the 129 patients with successful placement of ENBD; 125 (96.9%) had control of cholangitis within 1 to 3 days (Group A) or had successful prevention of cholangitis (Group B). Six of these patients did require repositioning of the catheter due to its being pulled out by the patient (n = 3) or getting kinked in the stomach (n = 3). Two patients had inadequate response and required additional percutaneous transhepatic biliary drainage. Remaining two patients died inspite of

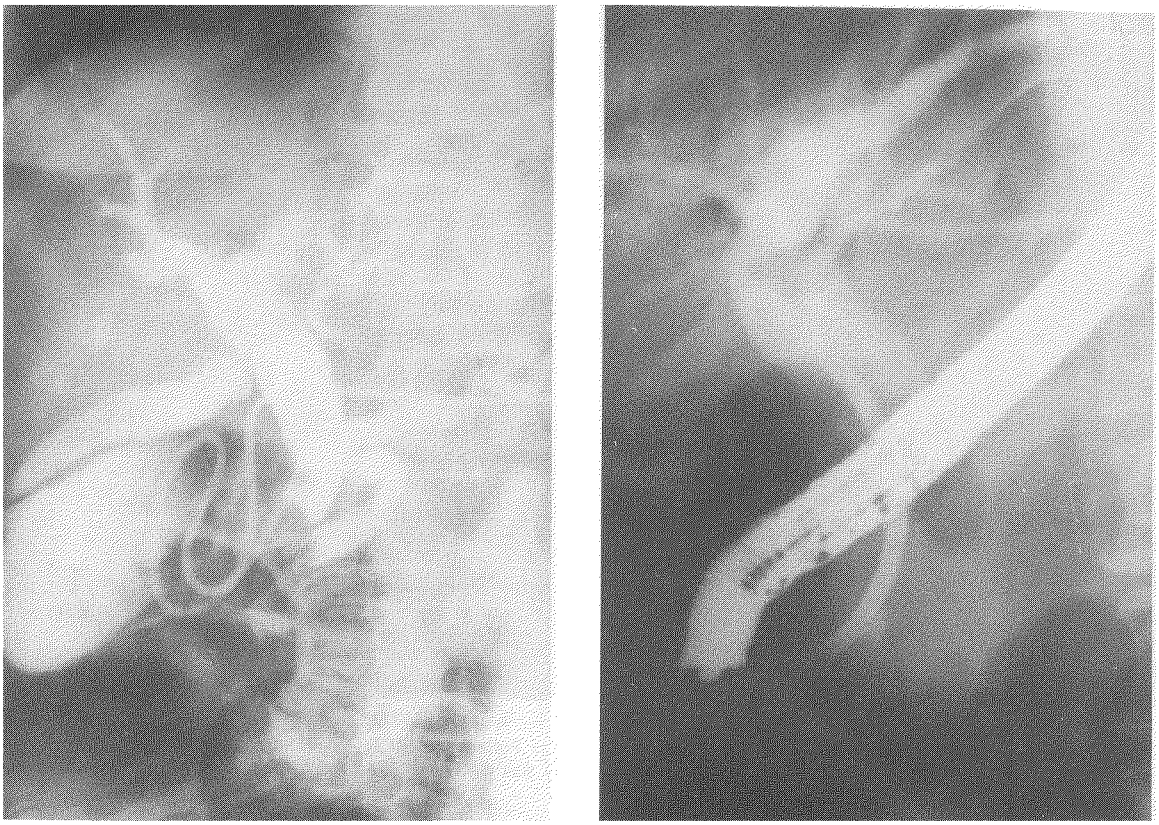

(A) (B)

FIGURE 2 Patient with ampullary carcinoma presented with cholangitis (a) Endoscopic nasobiliary drainage controlled the cholangitis (b) A 10 Fr stent was placed subsequently.

successful ENBD; one of these had a choledochal cyst and patient succumbed to septicemic shock within minutes of ENBD; the other patient had ENBD placed for malignant biliary stricture but had pulled out her catheter and died of septicemia before the catheter could be repositioned. In patients with favourable response to ENBD, catheter was removed after a period of 5 to 14 days (median: 8 days).

Of the surviving 127 patients with ENBD, 76 were given a more definitive treatment for the underlying biliary disease after stabilisation of patient's status. This included bile duct stone extraction after endoscopic sphincterotomy in 40 patients, biliary surgery in 28 and endoscopic biliary stent placement in 8.

### DISCUSSION

Endotherapy in acute cholangitis can be in the form of endoscopic sphincterotomy, biliary stenting or by nasobiliary drainage (ENBD). In the present series, we could establish ENBD in 90.2% of the patients and successful ENBD led to a rapid control of cholangitis or prevention of cholangitis in 97% of patients. The procedure was safe with no morbidity or mortality related to ENBD. The overall short-term mortality in our patient group was 3.5%, being 1.5% only in those with successful ENBD. Death was thus, always related to delay in institution of ENBD, displacement of catheter or failure to establish ENBD. Our results with ENBD are similar to the earlier reports with this procedure[8–13]. A

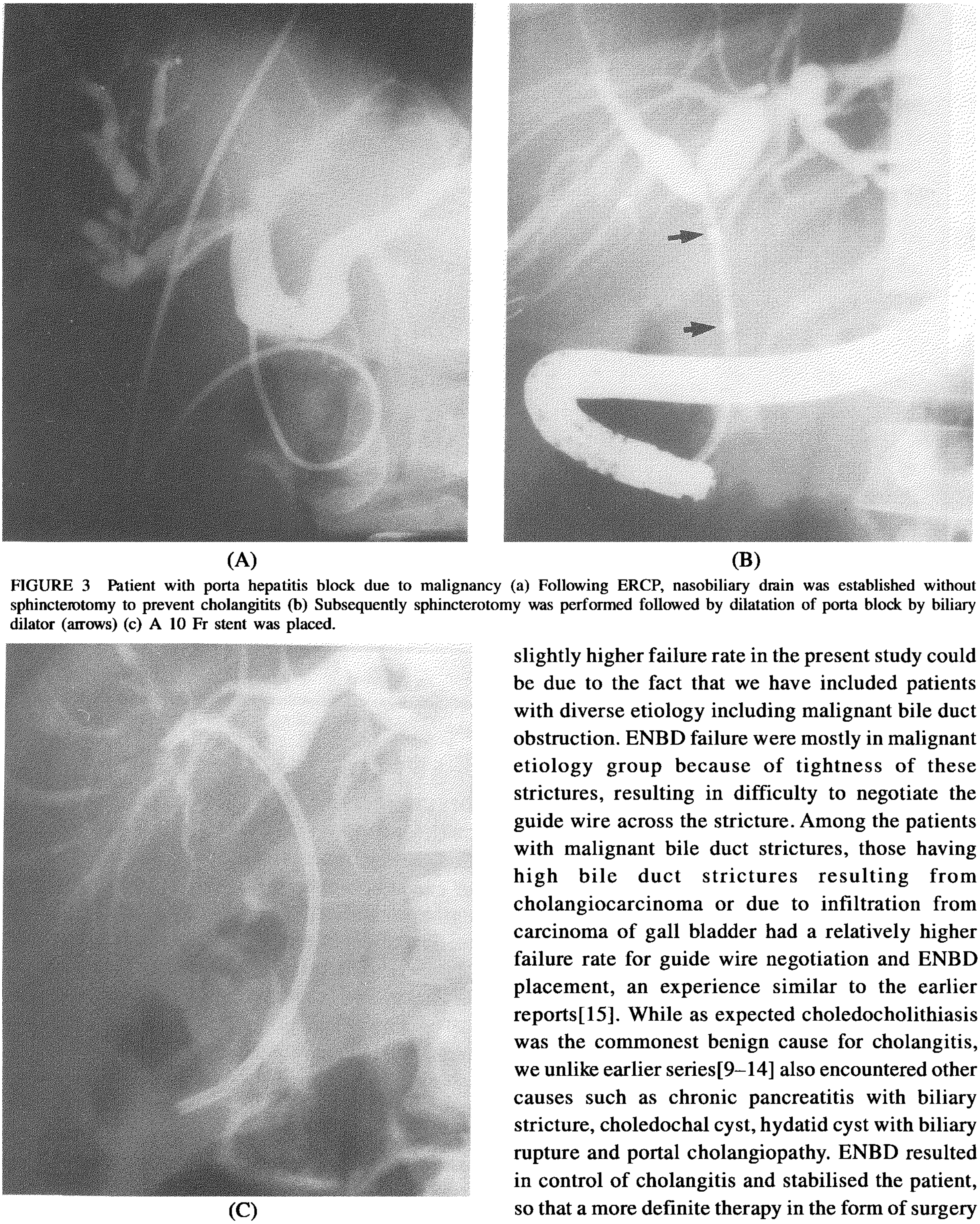

(A) (B)

FIGURE 3 Patient with porta hepatitis block due to malignancy (a) Following ERCP, nasobiliary drain was established without sphincterotomy to prevent cholangitits (b) Subsequently sphincterotomy was performed followed by dilatation of porta block by biliary dilator (arrows) (c) A 10 Fr stent was placed.

(C)

slightly higher failure rate in the present study could be due to the fact that we have included patients with diverse etiology including malignant bile duct obstruction. ENBD failure were mostly in malignant etiology group because of tightness of these strictures, resulting in difficulty to negotiate the guide wire across the stricture. Among the patients with malignant bile duct strictures, those having high bile duct strictures resulting from cholangiocarcinoma or due to infiltration from carcinoma of gall bladder had a relatively higher failure rate for guide wire negotiation and ENBD placement, an experience similar to the earlier reports[15]. While as expected choledocholithiasis was the commonest benign cause for cholangitis, we unlike earlier series[9–14] also encountered other causes such as chronic pancreatitis with biliary stricture, choledochal cyst, hydatid cyst with biliary rupture and portal cholangiopathy. ENBD resulted in control of cholangitis and stabilised the patient, so that a more definite therapy in the form of surgery

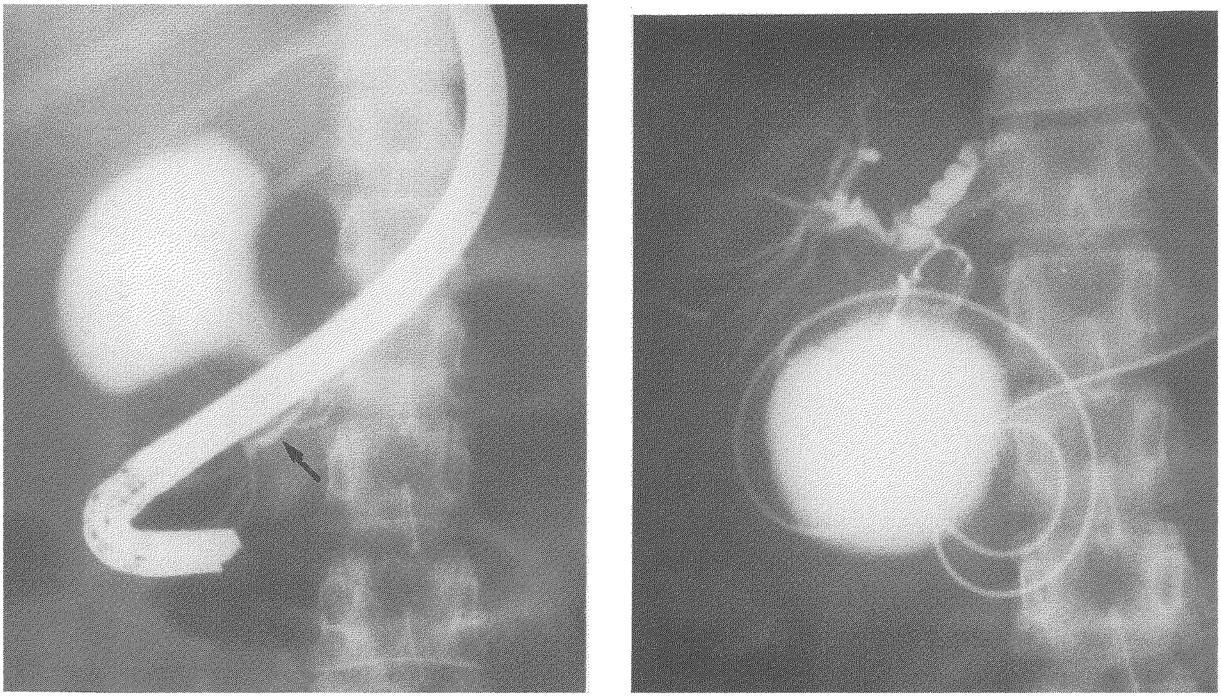

(A) (B)

FIGURE 4 (a) Patient with cholangitis had a choledochal cyst with a common channel (arrow) (b) Endoscopic nasobiliary drainage was established, cholangitis subsided.

or biliary stenting could be performed on a non-emergent basis.

Endoscopic sphincterotomy followed by dormia extraction in the same session is often considered the best therapy for cholangitis due to bile duct stones since it combines drainage of infected system along with removal of the cause[8–10]. However, endoscopic sphincterotomy in acute cholangitis has been associated in earlier reported series with complications such as hemorrhage, perforation and pancreatitis in 6–12% patients and mortality in 4.7–7.6%[8,10]. Moreover sphincterotomy may not always be possible in patients with cholangitis due to abnormal coagulogram, sphincterotomy combined with stone extraction by basket or balloon is more time consuming than ENBD placement without sphincterotomy[7,9,14] and ductal clearance after sphincterotomy has been achieved in earlier reports in 66 to 76% patients only[8–10]. In the present series, ENBD was performed without any prior sphincterotomy in 91.5% patients.

Some workers have preferred biliary stenting over ENBD while managing acute cholangitis because of patient's convenience with the former[16]. ENBD was preferred by us because the indwelling catheter permitted a cholangiogram to be performed later, allowing us to inject minimal required contrast material during initial procedure in the presence of infected bile. This reduces the risk of increased cholangiovenous reflux which can aggravate or precipitate septicemia in a patient with cholangitis[1,9]. ENBD moreover, allowed collection of bile for culture and flushing of catheter for clearing flakes of pus and bile debris. ENBD catheter can also act as a conduit for chemical dissolution of gall stones[1,14]. Though ENBD does carry the risk of being pulled out by the patient as happened in 3 of our cases, it can almost always be repositioned rapidly. Combining ENBD with stent placement in patients having cholangitis due to malignant biliary obstruction can cure cholangitis and at the same time provide the palliation for the tumor obviating the need for subsequent endoscopic

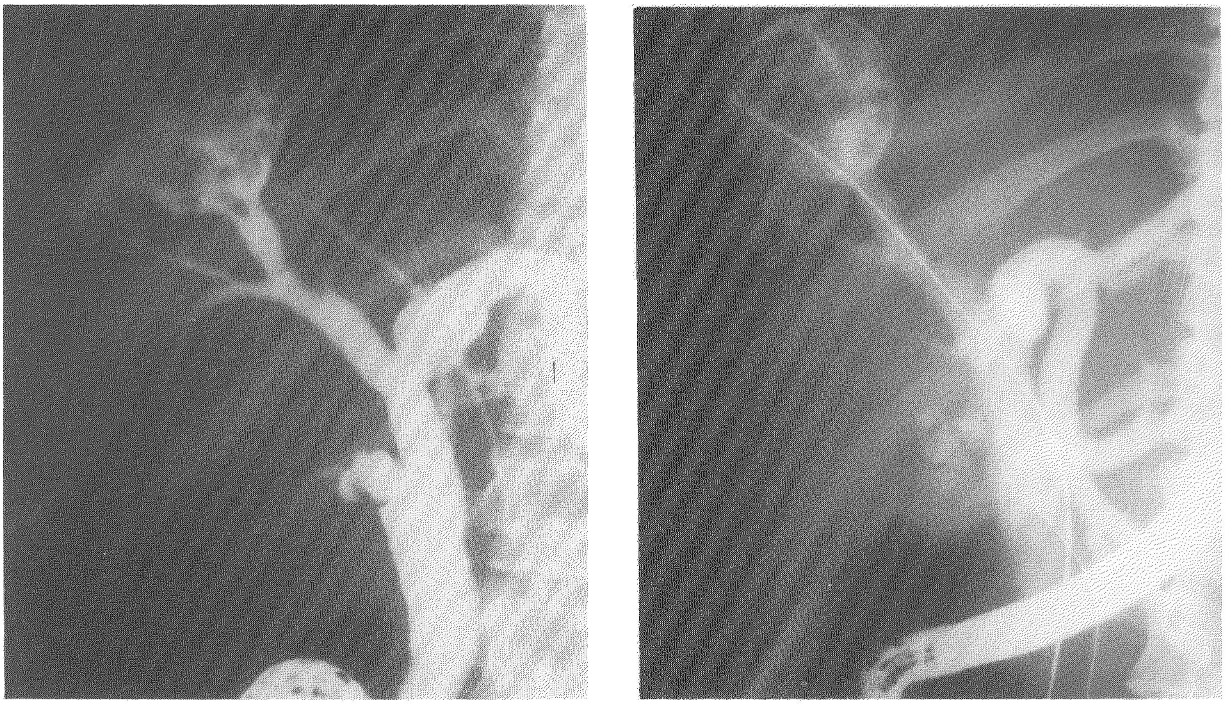

(A) (B)

FIGURE 5 (a) Patient with hepatic hydatid cyst with biliary rupture leading to cholangitis (b) Guide wire was passed into the cyst (c) Endoscopic nasobiliary drain resulted in control of cholangitits. Patient was operated later on an elective basis.

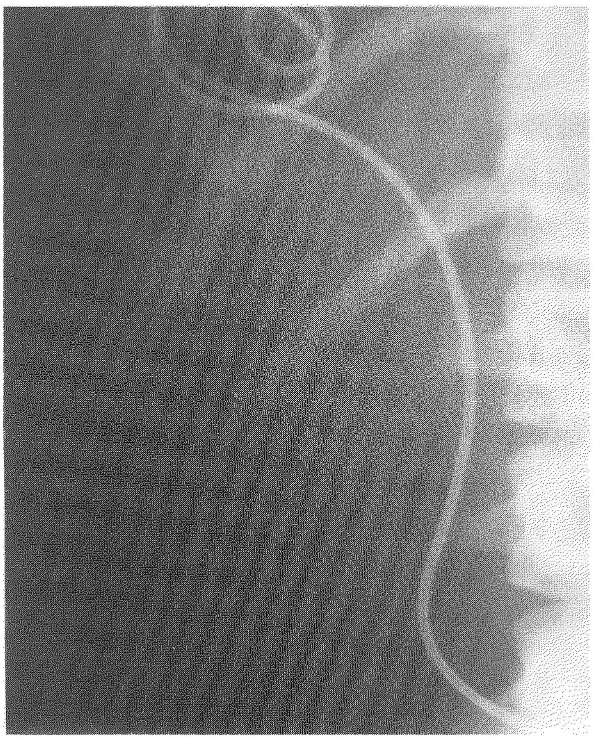

(C)

procedure. However, placement of two prosthetic device would require endoscopic sphincterotomy and a longer procedure time. We have preferred to treat the cholangitis in such setting by ENBD and a more definitive therapy by stenting or surgery was offered subsequently. Further, studies are however warranted to choose the optimum management modality in such a setting.

Percutaneous transhepatic biliary drainage (PTBD) and surgical treatment have been used earlier for treating patients with acute cholangitis. However, direct surgical intervention in acute cholangitis has been reported to carry a mortality of 6.5–40%[2–4]. PTBD is similarly associated with a significant mortality and a high risk (upto 28%) of bleeding, bile leakage and peritonitis and occasionally of pneumothorax, traumatic pseudoaneurysm and arterioportal fistula formation[5–7,17]. We therefore feel that surgery or PTBD should be performed only when ENBD is not possible due to anatomical reasons such as previous Polya's gastrectomy, is technically unsuccessful or rarely when ENBD does not improve the cholangitis.

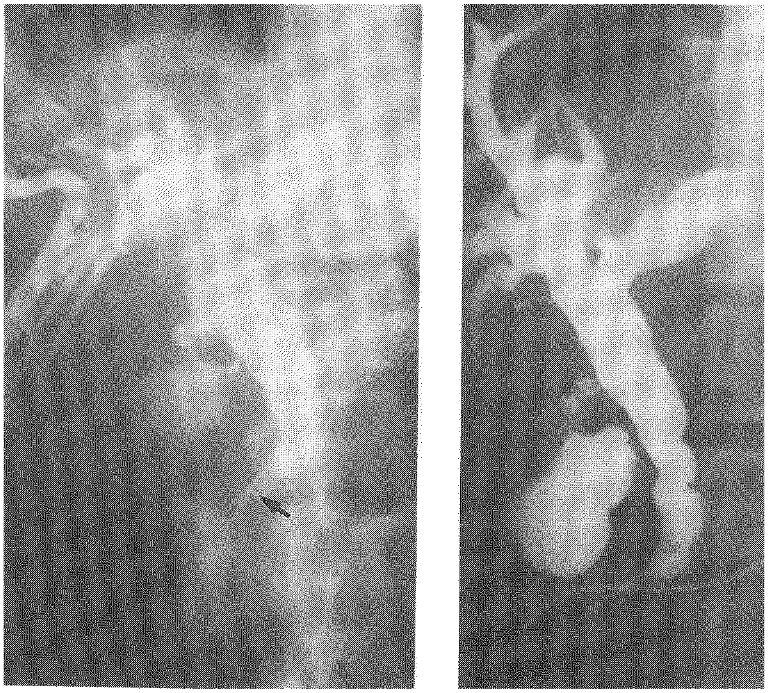

(A) (B)

FIGURE 6 (a) Patient with extrahepatic portal venous obstruction presented with cholangitis and was detected at ERCP to have stricture of lower part of bile duct (arrow). (b) Cholangitis subsided after endoscopic nasobiliary drainage.

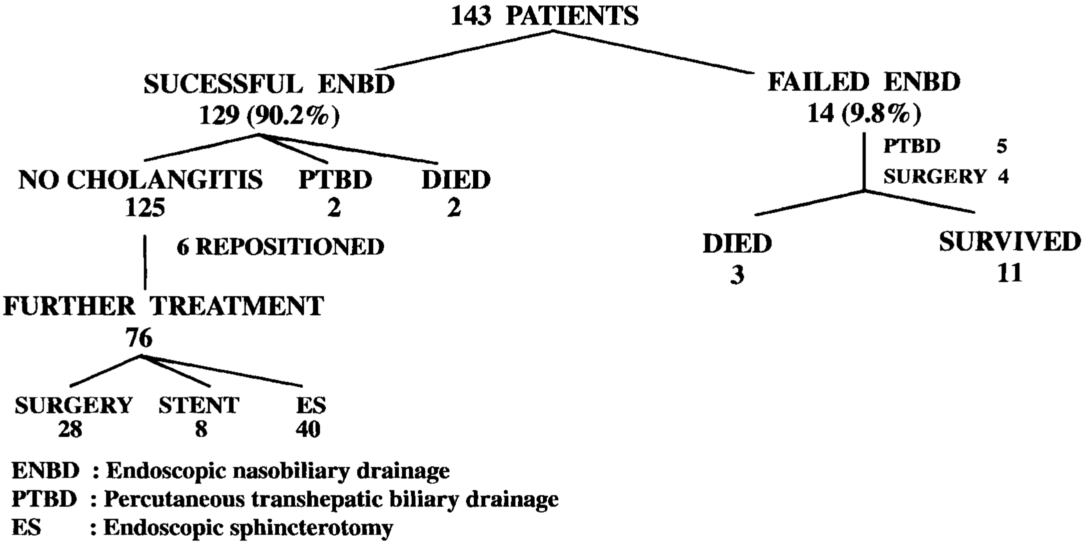

FIGURE 7 Outcome in 143 patients with attempted endoscopic nasobiliary drainage.

PTBD was performed in 7 patients in the present series, in 5 patients beuse of failed ENBD and in 2 with successful ENBD for more effective drainage.

In conclusion, the present study performed over a large number of patients and in a wide etiological spectrum confirms the usefulness and safety of ENBD in the treatment and prevention of acute cholangitis.

### *References*

[1] Leung, J.W.C. and Cotton, P.B. (1991). Endoscopic nasobiliary catheter drainage in biliary and pancreatic disease, *Am. J. Gastroenterol.*, **86**, 389–394.

[2] Lai, E.C.S., Tam, P.C., Paterson, I.A. *et al.* (1990). Emergency surgery for severe acute cholangitis: the high risk patients, *Ann. Surg.*, **211**, 55–59.

[3] Welch, J.P. and Donaldson, G.A. (1976). The urgency of diagnosis and surgical treatment of acute suppurative cholangitis, *Am. J. Surg.*, **131**, 527–532.

[4] Gigot, J.F., Leese, T., Dereme, T., Coutinho, J., Castaing, D. and Bismuth, H. (1989). Acute cholangitis: multivariate analysis of risk factors, *Ann. Surg.*, **209**, 435–438.

[5] Pessa, M.E., Hawkins, I.F. and Vogel, S.B. (1987). The treatment of acute cholangitis. Percutaneous transhepatic biliary drainage before definitive therapy, *Ann. Surg.*, **205**, 389–392.

[6] Kadir, S., Baassirri, A., Barth, K.H., Kaufman, S.L., Cameron, J.L. and White, R.I. (1982). Percutaneous biliary drainage in the management of biliary sepsis, *AJR*, **138**, 25–29.

[7] Gould, R.J., Vogelzang, R.L., Neiman, H.L., Pearl, G.J. and Poticha, S.M. (1985). Percutaneous biliary drainage as an initial therapy in the sepsis of the biliary tract, *Surg. Gynecol. Obstet.*, **160**, 523–527.

[8] Leese, T., Neoptolemos, J.P., Baker, A.R. and Car-Lockee, D.L. (1986). Management of acute cholangitis and the impact of endoscopic sphincterotomy, *Br. J. Surg.*, **73**, 988–992.

[9] Leung, J.W.C., Chung, S.C.S., Sung, J.J.Y., Banez, V.P. and Li, A.K.C. (1989). Urgent endoscopic drainage for acute suppurative cholangitis, *Lancet*, **1**, 1307–1309.

[10] Gogel, H.K., Runyon, B.A., Volpicelli, N.A. and Palmer, R.C. (1987). Acute suppurative obstructive cholangitis due to stones: treatment by urgent endoscopic sphincterotomy, *Gastrointest. Endosc.*, **33**, 210–213.

[11] Lai, E.C.S., Paterson, I.A., Tam, P.C., Choi, T.K., Fan, S.T. and Wong, J. (1990). Severe acute cholangitis: the role of emergency nasobiliary drainage, *Surgery*, **107**, 268–272.

[12] Lai, E.C.S., Mok, F.P.T., Tan, E.S.Y. *et al.* (1992). Endoscopic biliary drainage for severe acute cholangitis, *N. Engl. J. Med.*, **326**, 1582–1586.

[13] Chawla, Y.K., Sharma, B.C. and Dilawari, J.B. (1994). Endoscopic nasobiliary drainage in acute suppurative cholangitis, *Indian J. Gastroenterol.*, **13**, 83–85.

[14] Lin, X.Z., Chang, K.K., Shin, J.S. *et al.* (1993). Emergency endoscopic nasobiliary drainage for acute calculous suppurative cholangitis and its potential use in chemical dissolution, *J. Gastroenterol. Hepatol.*, **8**, 35–38.

[15] Huibregtse, K. and Tytgat, G.N.J. (1989). Palliative treatment of jaundice by transpapillary introduction of biliary endoprosthesis, *Gut*, **23**, 371–375.

[16] Kill, J., Kruse, A. and Rokkjaer, M. (1989). Large bile duct stones treated by endoscopic biliary drainage, *Surgery*, **105**, 51–56.

[17] Mueller, P.R., van Sonnenberg, E. and Ferrucci, J.T. (1982). Percutaneous biliary drainage: technical and catheter-related problems in 200 procedures, *AJR*, **138**, 17–23.

